# Gendered role disruption in women with rheumatoid arthritis: A phenomenological study in Kashmir^☆^

**DOI:** 10.1016/j.dialog.2026.100279

**Published:** 2026-01-11

**Authors:** Zakir Hussain Gadda, Mohmad Saleem Jahangir, Aneesa Shafi

**Affiliations:** Department of Sociology, University of Kashmir, Jammu & Kashmir, India

**Keywords:** Arthritis, Chronic illness, Gender, Kashmir, Loss, Role, Adaptive strategies

## Abstract

This study employed a descriptive phenomenological approach to explore the day-to-day social role experiences of women living with Rheumatoid Arthritis (RA) in Kashmir. Participants were recruited through purposive and theoretical sampling until data saturation was reached. The study included 15 women, aged 21 to 67 years, with a clinical diagnosis of RA. Most of these women participants were married homemakers. Data were collected via in-depth semi-structured interviews and analyzed with the help of Colaizzi's method. Results revealed that the challenges posed by RA, including pain, discomfort and progressive disability, have curtailed the patients' ability to fulfill their culturally assigned social roles. Such limitations increased their vulnerability to impaired well-being and a loss of identity and purpose. Furthermore, RA has increased patients' dependence on others, which diminished their perceived significance both within and beyond their familial boundaries. As a result, they have adopted various strategies to adapt to the changing demands and expectations associated with their social roles. The study findings emphasize the need for active family involvement in RA care to promote empathy and support. Moreover, healthcare providers need to address not only the physical aspects of RA, but also its under-recognized emotional, psychological, and sexual impacts to improve the quality of care.

## 1 Introduction

Rheumatoid Arthritis (RA) is a chronic, progressive, autoimmune disease marked by the inflammation of synovial joints, which causes intense pain, stiffness, and compromised joint mobility [1,2]. Its global prevalence is estimated to be 0.3–1% of the total adult population, which amounts to nearly 18 million sufferers [2]. RA spans demographic boundaries and is more prevalent in the age group of 40 to 60 years [3], and women are three times more likely than men to be affected by it [4]. Moreover, the prevalence of RA differs by geography, and certain regions are more vulnerable than others. In Europe, about 1 to 2% of the population has RA [5], while in the United States, 1.3 million adults suffer from it [6]. In India, around 1.3 crore individuals, representing about 0.5–1% of the total population, are suffering from RA [7]. In Kashmir, the accurate statistics for RA remain elusive, but the rheumatologists of the region believe that 1 to 2% of the population suffer from RA. In their study, Minia and Verma [8] found that out of 1738 patients who attended the Rheumatology department at SKIMS, 615 (35.4%) were diagnosed with RA, with females having a higher incidence (76.7%) than males (23.3%). However, this is likely an underestimate due to underreporting from individuals with mild symptoms who, as such, prefer not to approach healthcare facilities.

RA cannot be cured permanently, but timely diagnosis and treatment can help manage the disease well and reduce its burdens. In India, the societal norm of patriarchy and the existence of a pluralistic healthcare system often result in delayed diagnosis/treatment and increased reliance of patients on untrained/traditional providers [9]. Also, the pan-India studies reveal that there are significant gender-based disparities in India, with women particularly those from the vulnerable section facing a range of socioeconomic, cultural and logistic barriers in access to healthcare [10,11]. Data from the seventh round of NSSO (2017–2018) highlights a significant gender gap in healthcare utilisation throughout India with women reporting lower hospitalisation rates and higher unmet needs [12]. In the context of Kashmir, illness experiences like that of RA are largely shaped by deep-rooted structural inequities and patriarchy, which in turn cause systemic barriers to care. In many households, women need to be accompanied by a *mehram* (male guardian) or seek permission from male members before seeing a doctor. In certain instances, family finances are often prioritised for the health needs of male members, and women's pain/symptoms are overshadowed, or dismissed as exaggeration or a pretext to avoid household/agricultural work. Not only this, women, usually from the low-income families, at times ignore their own health needs and prefer to fulfill the health needs of other family members, particularly children [13] as a part of their caregiving responsibilities. These barriers are rooted in structural gender norms and compel women to hide their suffering, and avoid seeking professional healthcare until their condition becomes severe.

In Kashmir, access to rheumatological services is limited, which is detrimental particularly for the marginalized women who are often financially dependent and face multiple accessibility barriers in availing the needed care. Advanced treatments such as joint replacement surgeries are not available at the local level, which compel patients to travel outside the Valley of Kashmir at expensive costs. For routine outpatient department (OPD) visits, patients from rural areas need to travel to urban settings, which is both financially as well as physically burdensome for them. Also, the Pradhan Mantri Jan Arogya Yojana (PM-JAY), commonly known as Ayushman Bharat Youjna which provides insurance cover of rupees 5 lakh per year to each Kashmiri is not helpful for RA patients, as demonstrated by Gadda and Jahangir [13] in their study on pathways to arthritis care. This is because PM-JAY focuses on inpatient department care (IPD), while RA patients usually require long-term medication particularly on OPD basis which along with transportation costs is not covered under the scheme [13]. Consequently, RA patients particularly those from the low-income households are unable to get the needed care, and many patients turn to alternate treatments including self-medication and local medical shops. This often contributes to delay in diagnosis and disease progression among patients. Research reveals that when RA is not managed properly, it can lead to progressive joint deterioration, disability, diminished quality of life, comorbidities, and increased mortality rates [4,13]. Between 1990 and 2017, RA contributed to 3.4 million disabilities globally [14], and overall the mortality rate among patients with RA remains higher than that of the general population [15].

Apart from being primarily a physiological condition, RA can also be viewed as a significant biographical event [16] with severe socioeconomic and psychological consequences for patients, and their caregivers. These consequences may vary depending upon the social groups patients frequently interact with, such as family, friends, and healthcare professionals [17]. Usually, the RA patients experience extreme fatigue, immense pain and restricted joint mobility that impede their ability to carry out their social, occupational, and leisure activities on a day-to-day basis. They have limited capacity to fulfill their social roles including the role in family relationships and caregiving [18]. RA can also negatively impact the sexual lives of patients, particularly young women [19], and influence their motherhood experiences. It contributes to practical and psychological complexities during pregnancy and childcare [20]. These complexities stem from the functional disability associated with RA, which makes the everyday tasks and social role performance more challenging for them [21].

RA incurs substantial direct and indirect costs to patients and their households, such as treatment expenses, loss of productivity [3] and lowered income for being unable to perform their expected economic roles properly. This failure carries significant weight in societies characterised by patriarchal socioeconomic structure and can result in a profound loss of status and self-worth among patients. The RA patients often adopt sedentary lifestyles and over the course of their illness become increasingly dependent on other people [22] particularly family members. Consequently, they experience loss of self identity, deformed body appearances, lowered self-esteem and negative emotions of sadness, frustration, anger, guilt, and shame [18,23]. Despite these difficulties, proper social support can enable RA patients to effectively manage their condition, improve their quality of life [24] and adapt to the ever-changing nature of their roles.

So far as the literature on RA in Kashmir is concerned, it predominantly focuses on its medical/clinical aspects, while the subjective experiences of patients, especially in relation to their socio-cultural roles are largely missing. Given the fact that chronic illnesses such as RA can disrupt the structures of everyday life [16], it becomes imperative to study the experiences of those living with them. Therefore, the present study attempted to explore the day-to-day social role experiences of women living with RA in Kashmir and to unearth the repercussions of disrupted role performance on their identities. Additionally, RA being a lifelong disease with unpredictable progression, the study focussed on the coping strategies employed by patients to renegotiate their altered social roles**.**

## 2 Methods

### 2.1 Design

The underpinning philosophy and structure of our study were shaped by the descriptive phenomenology advanced by Edmund Husserl and Alfred Schutz. According to them, a phenomenon should be examined in the context of how it is actually lived and experienced [25]. Husserl emphasized the significance of experiences as acknowledged by human consciousness, and advocated for their scientific exploration [26], as they play a key role in shaping individuals' comprehension of their life world.

### 2.2 Ethics

This study is a part of the first author's PhD program focussing on healthcare inequities in Kashmir, for which ethical approval was granted by the Institutional Review Board (IRB), of the Government Medical College, Srinagar, J&K, India on 8 November 2024 (Reference number: IRBGMC-SGR/Socio/842). Ethical considerations are of paramount importance in qualitative research, particularly when engaging in face-to-face interviews with vulnerable groups, such as patients with chronic illnesses [27]. Consequently, the principle of respect for autonomy was carefully addressed, and the participants were informed in advance about the study's objectives and verbal consent was obtained from them. Verbal consent was found to be the most appropriate method to ensure participant comfort. This verbal consent was formalised with the help of a consent attestation form, which the researcher (the third author, who conducted the interviews due to the sensitive nature of the topic) signed after each participant showed her willingness to participate. Also, it was explicitly communicated that participation was voluntary, and participants could choose not to answer specific questions or withdraw their consent at any time during the study. To ensure confidentiality, participants were represented by identifiers (P1, P2, P3, and so forth) throughout the study. Interviews were conducted in private settings, with only the interviewer and the interviewee present. The audio-recordings were deleted permanently after the completion of data analysis and the ethical principles of non-discrimination and respect for intellectual property were taken into consideration to ensure fairness and uphold the integrity of the study.

### 2.3 Participants

The key consideration in recruiting participants for a phenomenological study is to ensure that they have experienced a particular phenomenon in all its aspects/dimensions [28]. Therefore, this study involved women diagnosed with RA in the region of Kashmir, a predominantly patriarchal society where traditional gender roles are deeply rooted in cultural/religious norms. Women are expected to perform caregiving, domestic, and reproductive roles and their identities are by and large tied to these roles. In consequence, any deviation from these expectations is often met with social judgment. These expectations are stronger in rural and far-flung areas, where women enjoy limited autonomy in decision-making. For instance, married women who are unable to conceive and adopt the motherhood roles are often blamed and stigmatised even when the infertility may lie with the male partner [29]. In line with the phenomenological tradition, purposive and theoretical sampling techniques were employed to recruit the participants. Purposive sampling was used to identify and select Kashmiri women diagnosed with RA for over a year and aged 18 years or older. Initially, participants were selected through personal contacts drawn from the researchers' social/professional networks and referrals from trusted acquaintances. In the meantime, new participants were chosen by theoretical sampling, which was informed by the concepts and relationships that largely emerged during the iterative process of data collection and analysis. To confirm the sample size, the idea of data saturation (the point at which no new data emerges in interviews) which occurred at the twelfth interview was followed. However, to validate thematic saturation, three more interviews were conducted, bringing the total number of participants to 15 (Table 1). This sample size is deemed justifiable in descriptive phenomenology, as supported by Polit and Beck, who considered a sample of 10 or more as adequate [30]. The sample shows variation in age and educational status and this heterogeneity strengthens the phenomenological aim of our study to explore the core/shared experiences of social roles across diverse lifeworlds. We bracketed these variables during analysis to focus on the commonalities of living with RA shared by all women, irrespective of their age or education.

**Table 1:** Characteristics of participants (*n* = 15).

ID	Age (years)	Education	Marital status	No. of children	Work status	Monthly household income (₹)	Disease duration (years)
P1	52	Illiterate	Married	2	Housewife	45,000	19
P2	57	Illiterate	Married	7	Housewife	35,000	6
P3	67	Illiterate	Married	4	Housewife	1,50,000	23
P4	27	PhD scholar	Single	–	Researcher	1,20,000	1
P5	53	Illiterate	Married	2	Housewife	10,000	12
P6	52	10th	Married	4	Housewife	60,000	8
P7	42	Graduation	Married	–	Housewife	15,000	9
P8	58	11th	Married	3	Housewife	80,000	10
P9	27	PhD scholar	Single	–	Researcher	90,000	3
P10	65	Illiterate	Widowed	5	Housewife	10,000	8
P11	60	Illiterate	Married	–	Housewife	2,00,000	20
P12	52	Illiterate	Married	4	Housewife	90,000	4
P13	40	Illiterate	Married	1	Housewife	6000	4
P14	39	Graduation	Married	1	Housewife	8000	3
P15	21	12th	Single	–	Student	10,000	2

Source: Fieldwork.

### 2.4 Instrumentation

Data were collected through a semi-structured interview schedule formed with the help of extant sociological and medical literature concerning RA, and from the detailed discussions with participants in the pilot phase of the study. The interview schedule had 2 sections. The first section focused on the participants' socioeconomic and demographic characteristics, while the second part contained a series of open ended questions about the experiences of living with RA, its impact on social roles, crisis, loss, support and role renegotiation.

### 2.5 Procedure

Data was gathered through semi-structured interviews in person and at a location and time decided by the participants themselves. Accordingly, 10 interviews took place at the participants' homes, 3 at a private orthopedic clinic and the remaining 2 at the University of Kashmir. Interviews were conducted in the local Kashmiri language from November 9, 2024 to January 15, 2025, and each interview lasted approximately an hour. With participants' permission, interviews were recorded with the help of a smart-phone. Also, the field notes were maintained to enhance data collection and to provide a rich context for analysis [31].

### 2.6 Analysis

The recorded interviews were transcribed verbatim and Colaizzi's approach which incorporates aspects of Husserl's phenomenology, was found to be the best fit for analyzing the data [32]. This included, data familiarization by reading transcripts multiple times, identification of significant statements, formulation of meanings, grouping meanings into themes, creation of a description for each theme, incorporation of themes into an all-inclusive description, and verification of the fundamental structure.

### 2.7 Trustworthiness

Given that the researchers and participants share a similar cultural background, we maintained a reflexive stance to manage our positionality. Bracketing, a key aspect of Husserlian phenomenology, was adopted to accurately represent the subjects' experiences. This minimised the researcher bias, and helped to enhance the confirmability of the study. Also, a reflexive journal was maintained to document analytical decisions and support self-awareness. Credibility was strengthened through investigator triangulation, where all researchers independently analyzed the data using the same method of analysis and resolved the discrepancies through discussion until a consensus was reached among them. To further enhance credibility, member validation which is critical to descriptive phenomenology was conducted with the three available participants who confirmed the accuracy of the findings.

## 3 Results

From data analysis, six overarching themes emerged, which included: daily disruption in household roles; role of a spouse and the related crisis; role as a mother, socializer and caregiver; altered economic role, work and productivity loss; interpersonal dynamics outside the family sphere; and role in decision-making.

### 3.1 Daily disruptions in household roles

The pain, stiffness, inflammation, and fatigue associated with RA have been reported to significantly hinder the participants' ability to perform everyday tasks such as cooking, cleaning, and managing household affairs. For them, all those tasks that require physical exertion, such as bending, climbing, standing, and lifting or carrying objects are difficult to perform and as such their domestic role performance gets severely impacted. As a result, they experience heightened stress, anxiety, feelings of guilt, and even inferiority complex as well. One participant vividly described her challenges as:

Due to RA, standing for extended periods, handling heavy objects, and moving around are quite difficult for me. Everyday tasks like cooking, cleaning, and doing laundry have become nearly impossible. I feel incapable, sad, and guilty. (P6).

Moreover, some participants faced marginalization, denial of access to resources and opportunities, and social exclusion due to their inability to fulfill domestic responsibilities. A participant while sharing her poignant experience, stated:

Every year, during winters, my daughter's family moves to Jammu to escape the severe weather in Kashmir. They used to take me along, and I would manage all their domestic chores. Now, due to RA, they do not take me there, as I am no longer productive for them. (P3).

Another woman who faced mistreatment within her own family narrated:

I am unable to assist my daughter-in-law in household chores. Recently, she shouted at me, and expressed that if she had known about my disease, she would have never married my son. It was disheartening. (P5).

The inability of RA women to fulfill their domestic roles was reported to have increased their dependence on others, which in turn reduced their autonomy, independence, and self-esteem, apart from increasing their concerns about their immediate caregivers. A participant expressed her dependency as:

RA limited my choices and made me a dependent. My daughter skips university classes to do housework. My husband also performs domestic tasks. This heightens my anxiety and reminds me of my limitations. (P1).

A woman, whose daughter left the school to care for her, shared her remorse:

RA restricts me from doing daily tasks. Sadly, one of my daughters dropped out from the school to care for me and handle the household responsibilities on my behalf. I feel guilty for her academic loss. (P2).

Traditionally, women in Kashmir have actively participated in domestic chores both as daughters and wives. However, for girls with RA, this role became limited, leading to increased stress and guilt among them. A girl participant stated:

Due to RA, I am unable to perform my domestic responsibilities as daughters usually do in our culture. I feel sad and guilty for living a sedentary life and for being dependent on my family for almost everything. (P15).

While most of the participants grieved on their diminished ability to perform domestic roles, a few reported to have developed adaptive strategies to stay engaged in their domestic life. Also, they have learned to accommodate RA in their lives. A participant stated:

RA is chronic and I cannot work like before. However, I have found peace in praying, reading the Quran and other books, cooking while sitting, talking to relatives and getting involved in small tasks. These activities make me feel worthy. (P4).

### 3.2 Role as a spouse and the related crisis

Almost all the married participants in our study described RA as an obstacle in fulfilling their reproductive/sexual roles, attending to their husbands' needs, and maintaining intimate bonds. Factors such as pain, limited mobility, physiological changes, and difficulties in post-intimacy bathing were cited as major contributors in their decreased interest in sexual activities. These factors made it uncomfortable, difficult, or even impossible at times for the participants to participate in intimate relationships with their partners. One participant articulated:

Pain, limited joint mobility, and inflammation hinder intimacy and the obligatory bathing after intercourse. Therefore, my desire for sexual activities is limited which affects my role as a spouse and my ability to meet the expectations of my husband. (P14).

Women facing hurdles in sexual activities often reported feelings of grief, sadness, and guilt. It negatively impacted their self-esteem, subjected them to stigmatization, and also strained their marital relationships. This is because engaging in sexual activity with a spouse is considered a fundamental objective of Muslim marriage and a crucial aspect of womanhood in Kashmir. A participant lamented:

My religion teaches me to be obedient and available to my husband. Sadly, due to RA, I often fail to meet his desires. All this instills in me the feelings of inadequacy as a wife. (P6).

Another woman with severe RA shared:

The odor of ointments I apply bother my husband. I am unable to maintain proper hygiene. For him, I am not the wife he once knew, as I fail to perform my spousal roles. He often avoids me, and it seems as if he is tired of me. I fear if I am still a perfect partner. (P3).

Some participants, particularly those with young husbands, struggled with the fear of being disliked or abandoned by their partners primarily due to their inability to fulfill the expected sexual roles. Women diagnosed with RA during their reproductive years reported that they were not interested in carrying pregnancies due to their limited mobility and future uncertainties. This further impacted their intimate bonds. A mother of two explained:

It is difficult to carry a pregnancy when one is barely able to walk or hold a small object. Therefore, after diagnosis, we stopped going intimate to avoid the challenges associated with pregnancy and childbirth. (P1).

RA also heightened apprehensions among unmarried participants about their future roles as wives, as they perceived physical fitness as a prerequisite for finding a partner in Kashmir. A concerned participant stated:

Living with RA is like being disabled. Who will marry me? Somehow if I get married, will I be able to perform the role of a wife? Will I be able to manage a pregnancy? To be honest, my future seems uncertain. (P9).

Despite these challenges, participants were completely reluctant to discuss their altered sexual roles openly with healthcare professionals or therapists. This reluctance was primarily due to the cultural taboo associated with sexuality in Kashmir. One participant described her reluctance as:

Discussing sexual life with an outsider, especially a male doctor is a taboo here. I also hesitated to discuss it with the doctor. Even the doctors I have consulted so far have never inquired about the impact of RA on my reproductive life. (P7).

Some RA women, with proper support from their husbands, have developed adaptive strategies for intimacy beyond intercourse, utilizing assistive devices and waiting for feasible times to accommodate their needs and limitations as partners. A participant highlighted:

Sexual activity in winter and in the morning and evening is nearly impossible due to high fatigue and impaired mobility. However, during summer or daytime, and with the usage of assistive devices such as cushions or pillows, it becomes manageable. (P13).

### 3.3 Role as a mother, socializer and caregiver

Participants reported that the physical limitations they face in view of RA made it difficult or even impossible for them to perform the tasks of lifting, playing, feeding, changing children's clothes, and carrying them. These tasks are more daunting for them particularly during flare-ups or times of increased pain. RA has also restricted mothers' ability to prepare children for school, arrange lunches, or participate in their school-related events. This resulted in reduced involvement and interaction of RA mothers with their children. These women, as such, reported that their children faced issues in socialization, schooling, nutrition, health, and overall well-being. Therefore, mothers with RA experienced heightened feelings of guilt, sadness, anger, and negative self-perception for being unable to care for their children properly. This distress resulted in a potential loss of their identity and purpose. This is because women in Kashmir consider motherhood as a vital component of their sense of self. A participant shared:

I could not cook in the morning, so my children often went to school without breakfast. Gradually my son developed a migraine. Also, I was unable to hold the nail cutter and my children were embarrassed in the school for having long nails. As a mother, I feel guilty for not meeting their needs properly. (P3).

Another woman, who missed her kids' regular vaccinations, lamented:

I got RA when my children were in primary school. My restricted mobility hindered their vaccinations/check-ups. Also, I was unable to participate in their school-related activities. I would vent my anger on them. I feel like I am a failed mother. (P1).

In Kashmiri culture, women also play a significant role in the socialization of grandchildren, but due to RA, they often fail to do so. A grandmother expressed:

Earlier, I would take care of three kids simultaneously. Now I am unable to pick-up a single kid on my own. I need someone to help me hold a kid, even for a few minutes only. I fail to socialize them the way I should do as a grandmother. I feel worthless and sad. (P5).

Several women highlighted that due to RA, they were unable to perform some rituals which are of great significance in Kashmiri culture. For example, a mother-in-law due to restricted mobility, could not unveil her daughter-in-law during her first visit to their home. She shared:

I could not perform the custom of *Mohar Tulen* (lifting off the veil of my daughter-in-law during her arrival) due to my illness. Instead, my daughter did that. However, it was perceived as a superstitiously inappropriate act by others. Now, anything untoward that happens to the couple is linked to my absence in that custom. (P10).

The limitations experienced by women with RA as mothers or caregivers significantly impact the mother-child relationships and overall family dynamics. Participants, as such, expressed the need for support and assistance from their husbands and other family members, prompting a significant shift away from traditional gender roles. A woman shared:

My husband had to work like a domestic helper/maid due to my illness. He alone socialized our children. Even today, he manages the household tasks alongside his job. Without him, our family would have collapsed. (P1).

One more woman who lost her ability to work stated:

Instead of me caring for my children and husband, they have been caring for me due to my limitations. As a result, one of my daughters left school to take over my role as a mother and caregiver in the family. I feel guilty for her sacrifice. (P12).

### 3.4 Altered economic role, work and productivity loss

Before RA diagnosis, many participants were actively contributing to their family income by participating in various economic activities, such as taking care of cows and selling milk, working in farm gardens and agricultural fields. However, the dysfunctions associated with RA have diminished their economic roles and their participation in income generating activities, which exacerbated their financial dependence on others. As a result, women with RA were unable to earn like before, which gradually compounded their stress, helplessness, negative self-esteem and reduced financial freedom. Moreover, their reliance on others for healthcare expenses apart from overall well-being has further added to their feelings of vulnerability. A woman described:

Earlier, I used to work actively in our farm garden, agricultural land, and orchards. Also, I would rear cows, sell milk and earn sufficient money. Now, I am unable to earn and am financially dependent on my family. I feel like I am a burden on them. (P2).

Another woman who lost her capacity to earn due to RA, expressed:

I used to collect leftover saffron petals from our land during harvest time and earn a handsome amount from that. Now, I am unable to perform any of my economic roles. Financially, I am no longer independent. My survival depends entirely on my family. (P3).

One more participant, whose family faced hardships in arranging money for her treatment, shared:

Earlier, I would contribute to the family income. Now my family members need to finance my exorbitant treatment costs which is quite difficult for them. We have halted the construction of our house due to my treatment expenses. All this frustrates me. (P14).

The dual economic burden of not earning and utilizing family income for treatment has facilitated the exclusion and abandonment of a few participants in our study. A 57-year-old woman narrated:

In our culture, sons look after their elderly parents. However, my only son abandoned us in view of my treatment expenses. He lives in a separate house with his family. Now our unmarried daughters take care of me and my husband. Where will we go after their marriages? I am worried. (P2).

Women employed outside their homes faced unique challenges in maintaining their economic roles. The limitations induced by RA were reported to cause stamina loss, absenteeism, and limited ability to carry out physical tasks effectively. Consequently, they experienced potential setbacks in their careers and a decline in their economic role performance. A woman who used to work in the private sector narrated:

In the office, I kept my ailment hidden from my colleagues, but as the disease worsened, I experienced frequent absenteeism. This led to skepticism among my coworkers. Also, my employers were not satisfied with my absenteeism/work. Finally, I had to quit. (P14).

A young participant in our study, who could not secure a job due to RA and related limitations, described her ordeal as:

After completing my master's degree, I could not get a teaching job in a private school. I was told that only those who are physically fit and healthy are given jobs in the private sector. (P9).

The determination to recover, staying positive, and following medical advice properly has enabled a participant to continue her research work in the university lab despite the challenges posed by RA. She narrated:

It was difficult to collect plant samples and conduct their cytogenetic analysis in the lab due to excruciating pain. At times, I felt that my life was over. However, despite all odds, I stayed optimistic and followed the doctor's advice properly. Thank God, my condition is better now. (P4).

The traditional home-based remedies were reported by some participants as effective, accessible and affordable alternatives in managing the disease on a day-to-day basis. A participant described her hope and proactive health behaviour as:

Medication, prayers/supplications, a healthy diet consisting of Kashmiri eggs, ghee and *patche ras* (bone broth), apart from light exercise helped me regain some strength. For me, every small improvement matters a lot. (P9).

Another participant who belongs to a very poor family commenting on her coping strategies and hope of recovery as:

Economically, the disease has drained us. It is now difficult for my family to afford my regular medical expenses. At times, my mother applies *nune aab* (salt water), *nune bah* (steam of salt water), *yekh* (ice cubes), and oils on my joints. These remedies often help me cope with inflammation/pain. (P15).

### 3.5 Interpersonal dynamics outside the family sphere

Participants expressed that the RA-induced physical limitations restricted their ability to travel, visit relatives, and participate in social gatherings on significant occasions such as birth, wedding, festivals, illnesses and death/mourning. They were unable to take part in the age-old tradition of exchanging gifts on these occasions. This limitation increased their feelings of guilt, disappointment, and a sense of incompetence. One woman shared:

On various occasions, I used to visit my relatives and neighbors along with gifts. Since my RA, I am unable to do so and reciprocate their kind gestures. It fills me with deep regret and reminds me of my disability. (P5).

Another woman who lost her vigor to participate in various occasions, expressed:

RA impacted my ability to join my loved ones both on their joyous and challenging times and maintain the same level of reciprocity in gift-giving. I now see myself incompetent in managing the relationships. (P6).

The absence of women's participation in certain occasions resulted in misunderstandings, resentment, and strained relationships with their relatives. Perceptions of neglect and indifference emerged, causing relatives to distance themselves from the patients and their families. A woman lamented:

Due to severe pain and restricted mobility, I could not attend the wedding ceremony of my niece. Since then, my sister's family has cut all ties with our family. She got hurt that I missed an important event in their family. (P2).

Women with RA are highly dependent on others for support and assistance and are aware of the unique challenges they experience due to their condition. On many occasions, the lack of specific facilities has been a potential source of discomfort and embarrassment for them. Therefore, many women preferred not to visit their relatives on various occasions to spare them from the burden of arranging separate accommodations/facilities such as providing a standing commode, chair, or a bed for them. They as such preferred not to be the cause of any inconvenience or disruption to the routine of their hosts. A woman demonstrated:

I need a chair to sit on, a bed to sleep, and an assistant to walk. It is tough to host a person with specific requirements like me. So, I prefer not to attend social gatherings to ensure my safety and not to bother my relatives. (P13).

Moreover, the fear of social stigma or being labeled based on their condition also barred some women from participating in social gatherings. A participant mentioned:

The doctor has advised me to eat while keeping my right leg straight. Earlier, while eating this way at my relative's home, I was labeled as disabled and a *‘boetch’* (glutton) by many. To avoid such hurtful stereotypes, I prefer not to go anywhere. (P12).

One more woman added:

When I walk, it seems clear that I have some disability. To avoid negative perceptions or stereotypes, I long ago stopped visiting my relatives on all occasions, particularly during mourning periods. (P11).

Many participants described a sense of withdrawal from their social circles primarily due to their inability to participate in social gatherings, which limited their in-person interactions. Consequently, they developed feelings of isolation and anxiety, despite using virtual means to communicate. A woman told:

Visiting relatives was a source of solace. Now, being unable to do so, I feel disconnected from my social network. This heightens my anxiety. I do interact with my loved ones via phone, but that is insufficient to maintain healthy bonds and feel joyous. (P10).

Another woman while commenting on her inability to join her relatives and acquaintances, on various significant occasions, held:

Due to my condition, I am unable to visit my loved ones. It increases my isolation, and I miss my past life. To cope with this, I have turned to praying and watching YouTube videos to relieve stress. (P8).

### 3.6 Role in decision-making

Women with RA face profound challenges in performing decision-making roles both within and outside their homes. This is due to their limited mobility and incapacity to carry out day-to-day tasks. Their limited physical interaction, increased dependence on others, and societal perception that they are incompetent and useless further diminish their participation in decisions they once actively made. These decisions included the management of domestic chores, financial matters, lifestyle choices, reproductive issues, socialization of children, and social interactions. As a result, they experience a decreased sense of self-esteem and belonging within their networks, and at times feel anxious, devalued, and useless as well. A woman who previously lived in a joint family where her limited abilities were getting balanced shared:

When my sisters-in-law observed my inability to perform routine tasks, they chose to dissolve our joint family, specifically to exclude me. My voice was unheard. They assumed control over all the family decisions. (P8).

Another woman who was unable to carry a pregnancy and currently lives without any child, described:

Soon after marriage, I was diagnosed with RA. Day by day, my condition worsened. So, I decided not to carry a pregnancy. My husband always opposed this decision, despite seeing my disability-like condition. Now, I endure domestic violence for being unable to conceive. (P7).

In Kashmir, parents in particular are important decision makers when it comes to their children's marriages. They visit various locations and interact with many people to make sure that the right spouses are selected for their children. However, some study women who serve as household heads found it difficult to actively participate in this process due to RA. As a result, they were unable to decide on their children's future marital prospects, which caused significant delay in their marriages. A 65-year-old widow stated:

Due to physical limitations, I am unable to find suitable matches for my two daughters. I am unable to visit prospective grooms' homes, seek advice from trusted persons, and make informed decisions regarding compatibility. Unfortunately, they have surpassed the marriageable age. (P10).

Moreover, the decline in women's role in decision-making authority has led to the loss of autonomy, alienation, and a sense of powerlessness among them. A participant who was unable to take care of herself and her husband lost all her property while seeking assistance from a family. She expressed:

My husband and I offered our property to a relative in exchange for our life-long care and accommodation. Initially, they showed a lot of care, but now everything is dictated by them. We are like mere beggars, confined in a solitary room. We feel powerless, alienated, and disheartened. (P11).

Another woman feared losing her children as they were influenced by the opinions of her sisters-in-law. She explained:

When I was bedridden, my sisters-in-law were deciding what my children should wear, eat, whom they should talk to, and where they should go. They were socialized against my will and taught to remain far from me. I seemed a mere stranger to them. (P8).

## 4 Discussion

To the best of our knowledge, this study marks the initial exploration of the social role experiences of women living with RA in Kashmir. In line with prior studies [33,34], our findings confirm and contextualize the well-established link between RA-related disability and the disruption of day-to-day social roles and activities among women. This disruption in social roles increased their dependence and psychological distress which involved the feelings of guilt, worthlessness and diminished self-esteem, as reported in earlier studies on arthritis [22,35]. In addition to these general consequences, our results demonstrate how the progressive inability to perform domestic roles due to RA contribute to mistreatment, denial of access to resources and social exclusion. These context-specific insights reveal the critical role of societal expectations in determining a woman's status and significance within her networks.

RA-induced limitations also impacted participants' abilities to fulfill their roles as mothers and caregivers. Challenges in managing practical tasks related to both younger and older children as reported in previous RA research [21,36] were profoundly felt by our participants. Our study however, reveals the wider, cultural-specific consequences that emerge when women fail to fulfill their parental roles. In line with a study conducted by Parton, Ussher and Perz [36] we found that the limited involvement of mothers in their children's lives had adverse impacts on their schooling, nutrition and wellbeing. This finding highlights that RA impacts not only the patients, but also their immediate family members as well. Our analysis connected these parenting difficulties to the core psychological burden (such as guilt, sadness, and the grief for earlier life) documented in the literature [20,37] on RA. This profound distress experienced by our participants for not being able to perform domestic/caregiving roles is rooted in their inability/failure to meet the rigid culturally established gender norms and expectations. These expectations intrinsically tie a woman's value to her domestic productivity. This finding, as such, provides a concrete illustration of social role theory, by demonstrating how societal expectations can shape self-worth, and how the inability to fulfill social role obligations, as seen among participants, can disrupt an individual's sense of identity and purpose.

Most participants upon diagnosis preferred not to have children which impact their sexual and reproductive roles. This impact was compounded by a shared patient-provider reluctance to discuss sexual health, which is largely rooted in the cultural taboos surrounding sexuality in Kashmir. This finding reflects and extends a previous observation that conservative Asian norms can suppress open dialogue about sexual issues [22]. Crucially, our findings demonstrate how these taboos on sexuality exacerbate the psychological distress documented in the available RA literature. The unaddressed sexual and reproductive complexities intensified participants' feelings of guilt, sadness, distress, and inadequacy as partners, thereby confirming and contextualizing earlier reports of such emotional burdens [19,33]. Furthermore, the strain these sexual limitations placed on marital relationships strongly aligns with and reinforces the work of Josefsson and Gard [38], who noted that the women's inability to perform sexual roles can lead to marital breakdown.

The economic roles of our participants were severely disrupted by RA, which resulted in productivity loss, financial dependence on family members, and increased distress. This finding substantiates and contextualizes the more general observations about RA and work disability, like those made by Chorus et al. [39]. However, our data reveals that in the patriarchal framework of Kashmir, the consequences of economic role disruption extend beyond unemployment to a systematic loss of patients' social status and identity. In addition to the strain placed by role disruption on finances, the dual burden of income loss and treatment costs lead to the social exclusion and neglect of women. Their diminished importance within the family was largely justified by their increased financial dependence. Thus, while RA can generally result in work disability, in the particular context of Kashmir, the economic role disruption related to it can turn arthritis into a source of social marginalization.

The stigmatization faced by our participants provides a context-specific illustration of Goffman's theory, by demonstrating how RA-related disability can become a discrediting trait and result in patients' social disqualification. This stigma was particularly manifested in patients' exclusion from rituals and social exchanges. As a result, they experience guilt feelings, isolation, loss of interaction, and the weakening of kinship bonds. This pattern resembles with and deepens the findings of a study conducted by Fyrand et al. [40] on the disruption of social networks in RA. Our study links the social role disruption and related stigma to a loss of women's autonomy and voice in decision-making. This loss is a consequence of patriarchal power structures that restrict agency from women when they are perceived as no longer able to fulfill their expected socio-cultural roles. These findings elaborate the previous researcher by highlighting that the systematic disempowerment related to chronic diseases such as RA is a critical social justice issue that needs greater attention in social/health policy.

The coping strategies adopted by our participants, ranging from acceptance of RA as a lifelong ailment to the use of assistive devices, resemble the adaptive resilience reported in similar cultural settings like that of Odisha, India [9]. Our analysis also elaborated the established evidence on the pivotal role of social support by demonstrating its dual effect in the context of Kashmir. For instance, adequate family support, particularly from husbands, facilitated role renegotiation and coping, while its absence as highlighted by Kool and Geenen [41] led to a significant deterioration in patients' health/well-being and exacerbated their everyday challenges. Therefore, social support functions as a non-negotiable buffer against the consequences of social role disruption highlighted in the study.

Overall the participants' experiences documented in this study transcend the individual hardship and reflect a broader social justice issue. These findings demonstrate how systemic gender inequities increase the suffering of women living with RA in Kashmir. This supports the biopsychosocial understanding of illnesses, by exploring how biological symptoms (such as pain and fatigue) trigger psychological distress, which is then compounded by culturally enforced gender norms. This fact related to arthritis in Kashmir is consistently observed in the RA literature across different contexts. These structural inequities effectively transform the medical condition of RA into a heavy social burden of marginalization and disempowerment. Findings like this are predicted by theories that link chronic illness such as RA to social disqualification and the loss of active social role performance. Consequently, the suffering of women with RA is not a natural outcome of the disease alone, but is significantly produced and intensified by a social order that systematically devalues their well-being when they fail to meet the narrowly defined gender roles. This finding reinforces the core argument of the broader social-role literature in rheumatology.

This study provided valuable insights about the ways structural gender disparities interact with chronic illness. However, it is important to acknowledge its limitations, which include the small sample size and its exclusive focus on Kashmiri-speaking Muslim women, which limits the generalizability of its findings. This homogenous sample may also limit the understanding of how RA-related experiences might vary across multiple ethnic/religious groups residing within the region. Moreover, the study results are based on a particular cultural context of Kashmir, and hence they might not be directly applicable to societies with more accessible/affordable healthcare, different family-related expectations and greater gender-based equality/freedom. In such contexts, the experience of social role disruption may manifest itself differently. To address these limitations, further research with a larger and more socio-culturally diverse sample is required. Also, future studies could employ a comparative approach to explore how the demographic factors of age or education might influence the shared experiences of living with arthritis.

## 5 Conclusion and implications

In conclusion, women in Kashmir perform diverse roles as homemakers, spouses, mothers, caregivers, wage earners, and decision makers to name a few. However, the challenges and progressive disability associated with RA have curtailed their ability to perform these social roles, leaving them vulnerable to impaired well-being and a potential loss of identity and purpose. Most of our participants were diagnosed with RA in their highly productive years, and as the condition worsened with time, they began to perceive themselves as inadequate to fulfill their social role obligations. Consequently, RA has heightened their dependence on others and over time they experienced a loss of significance within their own families and other social spheres. Therefore, women with RA adopted different coping strategies, including disease acceptance, reliance on religion, and seeking assistance from supportive family members.

The study findings emphasize the importance of promoting active family involvement in patient care to enhance empathy and support throughout the illness experience. There is a need to organise educational programs/workshops at the family/village level to disseminate awareness regarding the impact of RA on social roles, and reduce stigma through enhanced empathy. Also, the healthcare providers need to respond sensitively not only to the physical aspects of RA, but also to its usually unspoken emotional, psychological and sexual consequences in order to improve the quality of care. Healthcare professionals should integrate the psychosocial and sexual health assessments into routine RA care and promote culturally-sensitive spaces for discussion on these unspoken issues. The impact of RA on women's economic participation including the loss of livelihood demands attention from voluntary organizations, care professionals, and society at large, to improve their quality of life and timely access to treatment. For policymakers in particular, these results highlight the need for social protection schemes that address the financial burden of treatment costs and income loss faced by marginalized patients.

## CRediT authorship contribution statement

**Zakir Hussain Gadda:** Writing – original draft, Resources, Methodology. **Mohmad Saleem Jahangir:** Writing – review & editing, Supervision, Software, Conceptualization. **Aneesa Shafi:** Formal analysis, Data curation.

## Ethical statement

Ethical approval was granted by the Institutional Review Board (IRB), of the Government Medical College, Srinagar, J&K, India (Reference number: IRBGMC-SGR/Socio/842). Also, informed consent was obtained verbally from each participant and then formally documented with the help of an attestation form. Participants' right to voluntary participation, and privacy were strictly upheld.

## Declaration of competing interest

The authors declare no potential conflicts of interest with respect to the research, authorship, and/or publication of this article.
